# Purinergic System Signaling in Metainflammation-Associated Osteoarthritis

**DOI:** 10.3389/fmed.2020.00506

**Published:** 2020-08-28

**Authors:** Paula Gratal, Ana Lamuedra, Juan Pablo Medina, Ismael Bermejo-Álvarez, Raquel Largo, Gabriel Herrero-Beaumont, Aránzazu Mediero

**Affiliations:** Bone and Joint Research Unit, IIS-Fundación Jiménez Díaz UAM, Madrid, Spain

**Keywords:** purinergic system, A2AR, P2X7 receptor, osteoarthritis, metainflammation, mitochondrial metabolism, Inflammasome, rheumatic diseases

## Abstract

Inflammation triggered by metabolic imbalance, also called metainflammation, is low-grade inflammation caused by the components involved in metabolic syndrome (MetS), including central obesity and impaired glucose tolerance. This phenomenon is mainly due to excess nutrients and energy, and it contributes to the pathogenesis of osteoarthritis (OA). OA is characterized by the progressive degeneration of articular cartilage, which suffers erosion and progressively becomes thinner. Purinergic signaling is involved in several physiological and pathological processes, such as cell proliferation in development and tissue regeneration, neurotransmission and inflammation. Adenosine and ATP receptors, and other members of the signaling pathway, such as AMP-activated protein kinase (AMPK), are involved in obesity, type 2 diabetes (T2D) and OA progression. In this review, we focus on purinergic regulation in osteoarthritic cartilage and how different components of MetS, such as obesity and T2D, modulate the purinergic system in OA. In that regard, we describe the critical role in this disease of receptors, such as adenosine A2A receptor (A2AR) and ATP P2X7 receptor. Finally, we also assess how nucleotides regulate the inflammasome in OA.

## Introduction

The biological actions of purine nucleotide and nucleoside signaling have been recognized since 1929. ATP was proposed as responsible for non-adrenergic, non-cholinergic intestinal and bladder transmission. However, it was not until 1972 that *G*. *Burnstock*, the founding father of purinergic scientific research, introduced the term “purinergic signaling” (1) and in 1976 specific receptors for extracellular nucleotides were defined (2).

**Figure 1:**
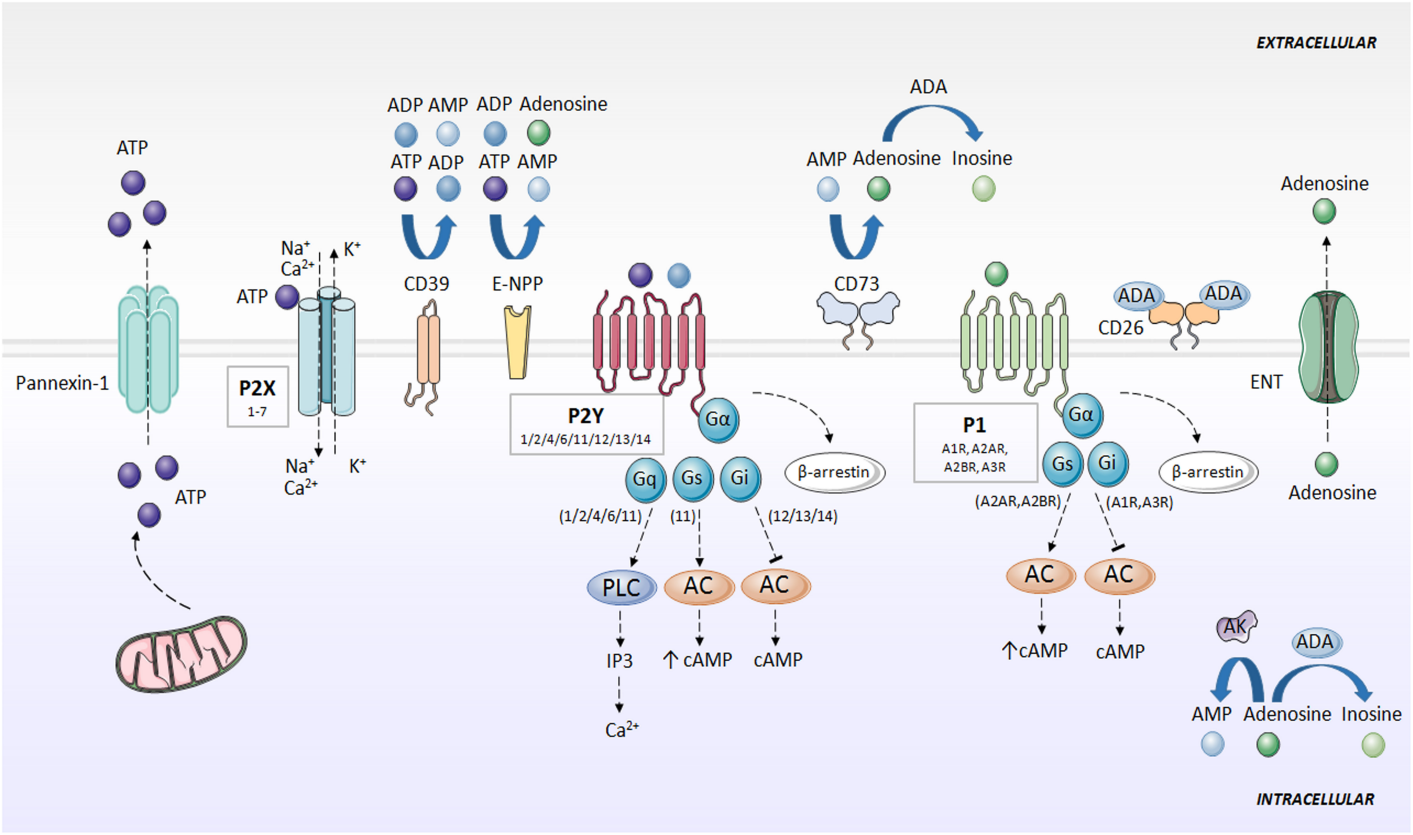
Purinergic signaling and specific receptors for extracellular nucleotides. ATP is released to the extracellular space mainly through pannexins (e.g., Pannexin-1). Then, ATP is rapidly degraded to ADP, AMP, and adenosine by the action of CD39, E-NPP and CD73. Adenosine can be converted to AMP through AK action, or degraded to inosine by ADA. Adenosine is also directly released outside the cell through ENT. P2X receptors activated by ATP, forming an ion channel for Na^+^, K^+^, and Ca^2+^. P2Y receptors are activated by ATP and ADP, and associate with G proteins, promoting intracellular second messengers' cascades, including Ca^2+^ and cAMP. Adenosine activates P1 receptors, coupled to G-proteins. A1R and A3R interaction with Gi, and A2AR and A2BR with Gs. P1 and P2Y receptors desensitized through internalization by β-arrestins. AC, adenylate cyclase; ADA, adenosine deaminase; ADP, adenosine diphosphate; AK, adenosine kinase; AMP, adenosine monophosphate; ATP, adenosine 5′-triphosphate; cAMP, cyclic adenosine monophosphate; CD26, dipeptidyl peptidase IV; CD39, ecto-nucleoside triphosphate diphosphohydrolase; CD73, ecto-5′-nucleotidase; E-NPP, ecto-nucleotide pyrophosphatase/phosphodiesterase; ENT, nucleoside transporter; G, G protein; IP3, inositol trisphosphate; P1, adenosine receptors; P2X, ionotropic nucleotide receptors; P2Y, metabotropic nucleotide receptors; PLC, phospholipase C.

ATP is known for being the universal energy currency. An equilibrium between the intracellular and extracellular amount of ATP is maintained in basal conditions, but in certain physiological and pathological situations, such as apoptosis, infections, mechanical stress, and inflammation (3), cells release ATP from intracellular deposits to the extracellular space. This process is mediated by pannexin (e.g., Pannexin-1) (4) and connexin hemichannels (e.g., Connexin-43) (5–7), but also other ion channels, such as calcium homeostasis modulator 1 (CALHM1) (8), volume-regulated anion channel (VRAC) and maxi-anion channel (MAC) (9), vesicular exocytosis (10) and autophagy-dependent lysosomal exocytosis (11, 12), and through uncontrolled release in apoptotic processes (3). In the extracellular compartment, ATP has an entirely different function and activates purinergic signaling via ion channel and transmembrane purinergic receptors in the cell membrane (3) (Figure 1).

ATP is rapidly degraded to adenosine, which also exerts an important role in purinergic signaling. Outside the cell, several enzymes hydrolyze ATP and limit nucleotide availability for purinergic signaling. Ecto-nucleoside triphosphate diphosphohydrolase (CD39) converts ATP into ADP, and ADP into AMP. Ecto-nucleotide pyrophosphatase/phosphodiesterase (E-NPP) also converts ATP directly into AMP and hydrolyzes ADP directly to adenosine. Separately, ecto-5'-nucleotidase (CD73) transforms AMP into adenosine (13–16). Additionally, one of the main regulators of the purinergic system in the extracellular space is adenosine deaminase (ADA), which anchores to the plasma membrane through the dipeptidyl peptidase IV (CD26) (17) and metabolizes adenosine to inosine (7, 18). Adenosine kinase (AK) converts adenosine back to AMP (19). Adenosine can also be directly released outside the cell through nucleoside transporters (ENT) (18) (Figure 1).

Pharmacology recognizes two families of purinergic receptors: P1 receptors, selective for adenosine (20), and P2 receptors, selective for nucleotides and dinucleotides of purines and pyrimidines (21).

P1 or adenosine receptors are G-protein coupled receptors (GPCR) currently divided into four subtypes, named A1R, A2AR, A2BR, and A3R, whose activation is dependent on the presence of extracellular adenosine (20). A1R and A3R differ from A2AR and A2BR in the particular G protein they interact with, with A1R and A3R being inhibitors (coupled to Gi), and A2AR and A2BR promoters (coupled to Gs) of cAMP synthesis via adenylate cyclase (AC). The modulation of cAMP levels controls multiple signaling pathways, including mitogen-activated protein kinase (MAPK) and serine-threonine specific kinases (22). Adenosine receptors are desensitized via through their internalization by β-arrestins (18, 23) (Figure 1).

Within the P2 or nucleotide receptors, P2X are ionotropic and P2Y are metabotropic receptors. P2X receptors are activated by ATP, and seven subtypes (1–7) have been identified. P2X receptors are homo- or heterotrimers, which combine differently (e.g., P2X2/3, P2X1/2, P2X1/5, P2X2/6, P2X4/6, and P2X1/4) and form an ion channel for Na^+^, K^+^ and Ca^2+^ (18, 23). P2Y receptors (P2Y1/2/4/6/11/12/13/14) are selective for ATP, ADP, UTP, and UDP, and couple with G proteins (21). Like adenosine receptors, P2Y receptors are internalized by β-arrestin, resulting in desensitization of the purinergic signal (18, 23, 24) (Figure 1).

Purinergic signaling is evolutionarily conserved (25), and is involved in several physiological and pathological processes, such as neurotransmission, cell proliferation, platelet aggregation, vasodilatation, and inflammation (23, 24).

Purinergic signaling contributes to the pathophysiology of several bone and cartilage diseases, such as OA, rheumatoid arthritis (RA), and osteoporosis (18).

Osteoarthritis (OA) is the most frequent and most disabling rheumatic disease in developed countries (26, 27). Around 250 million people suffer from knee OA worldwide, with a 33% prevalence in the population aged over 65 years, and with women being more affected than men (28, 29). OA is characterized by the progressive degeneration of diarthrodial joints (30), involving all the joint tissues, especially articular cartilage (AC), which suffers erosion and progressively becomes thinner. OA has traditionally been considered a “wear and tear” disease, caused by mechanical cartilage breakdown. However, it is now well-accepted that inflammation plays a critical role in the disease's progression both in cartilage and the synovium (31). Among the multifactorial etiology of OA, metabolic syndrome (MetS) and aging are critical risk factors for the onset of the disease, due in part to a state of low-grade chronic inflammation (32). The interaction between tissue damage and destruction by mechanical and biological mechanisms, together with the activation of innate immunity by multiple local and systemic inflammatory factors, are responsible for the chronicity of pathological processes in OA.

In this review, we present the important contribution of MetS and purine metabolism to the chronic pro-inflammatory status during OA progression. Moreover, we provide an integrated view of the mechanisms triggered by low-grade chronic inflammation as a pivotal axis in the pathogenesis of OA.

## Metainflammation in OA

MetS is an increasingly prevalent condition, generally diagnosed when more than two of the following risk factors are present: central obesity, lowered HDL cholesterol, high triglycerides, hypertension, and/or impaired glucose tolerance (33–35).

Metainflammation, the inflammation triggered by a metabolic imbalance (36), is a low-grade inflammation generated by components implicated in MetS. This phenomenon is mainly caused by excess nutrients and therefore, by an energy surplus, and it contributes to the pathogenesis of OA (37).

Recent studies have focused on identifying the relationship between MetS components and OA in order to understand its influence on the inflammatory process involved in the pathogenesis of the disease. An epidemiologic association between MetS and OA has been observed in clinical studies, especially with knee OA (38). Animal studies have also demonstrated that OA pathogenesis can be led by metabolic dysregulation (38). Despite increasing evidence of an association between OA and MetS, the mechanisms linking these diseases are not fully understood (39).

### Obesity and Metainflammation in OA

Central obesity may lead to OA pathogenesis by promoting systemic and local inflammation and by increasing load and consequent mechanical wear of the joints (40). There is an association between obesity and OA both in weight bearing and non-weight bearing joints, which highlights the key role of chronic inflammation in the disease's development (41).

**Figure 2:**
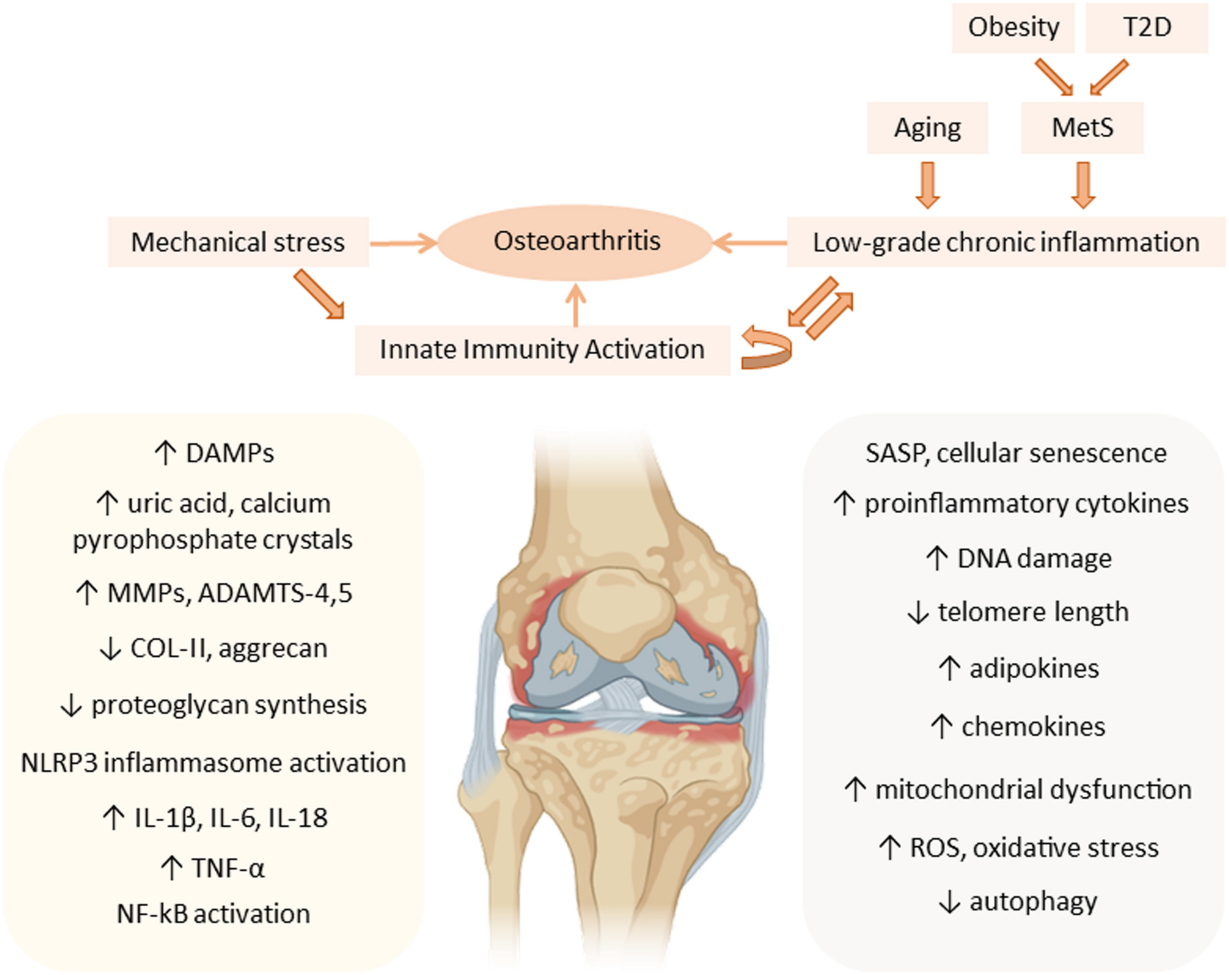
Metainflammation in the pathogenesis of osteoarthritis (OA). Mechanical stress contributes, together with the low-grade chronic inflammation associated with aging and the components of the metabolic syndrome (MetS), to the chronic activation of innate immunity in the joint, mainly affecting articular cartilage, during OA progression. Aging-associated factors and senescence together with metabolic factors, such as an increase in adipokines, maintain a systemic low-grade chronic inflammatory status that contributes to the pathogenesis of OA, increasing the expression of cartilage catabolic enzymes (MMPs, ADAMTS), and pro-inflammatory cytokines (IL-1β, IL-6, IL-18, and TNF-α), thus perpetuating inflammation in the joint. ADAMTS, a disintegrin and metalloproteinase with thrombospondin motifs; COL-II, type II collagen; DAMPs, danger-associated molecular patterns; DNA, deoxyribonucleic acid; IL, interleukin; MMP, matrix metalloproteinases; MetS, metabolic syndrome; NF-kB, nuclear factor kappa B; NLRP3, NOD-, LRR- and pyrin domain-containing protein 3; ROS, reactive oxygen species; SASP, senescence-associated secretory phenotype; T2D, type 2 diabetes; TNF, tumor necrosis factor.

Increased adipose tissue on the body exerts metainflammation through the production of adipokines and cytokines (42, 43). Some secreted adipokines are leptin, resistin and adiponectin (44). They are able to modulate the immune system and induce synthesis of pro-inflammatory and catabolic mediators, which leads to chondrocyte dysfunction and aggravates OA progression (45). These adipokines are distributed systemically and can be found in high amounts in osteoarthritic synovial fluid (45). They are synthesized by synoviocytes, articular chondrocytes and adipocytes of intra-articular fat tissue (37) (Figure 2).

Leptin plays an essential role in energy metabolism, leading to energy consumption. It affects growth factor synthesis and anabolism, and it is present in human chondrocytes, synovium, osteophytes and infrapatellar fat pads (44). In OA, this protein shows a marked expression in cartilage and osteophytes in comparison with healthy tissue (46). Furthermore, leptin contributes to the pathogenesis of OA, since it has a pro-inflammatory and catabolic role in cartilage metabolism, its expression being directly associated with the degree of cartilage destruction, and through the stimulation of growth factor synthesis (46, 47). Leptin induces the synthesis of matrix metalloproteinases (MMPs) in primary cultures of human chondrocytes. Accordingly, in patients with OA its concentration correlates with MMP-1 and MMP-3 levels in the synovium (48, 49). Furthermore, leptin promotes the activation of monocytes, and a subsequent increase in macrophages in the synovium (32).

Resistin is mainly expressed in white adipose tissue, but also by macrophages, osteoblasts, osteoclasts, and chondrocytes (40). Resistin is capable of inducing cytokine and chemokine expression in chondrocytes (50), being a potential regulator of the pro-inflammatory cytokines that activate transcription nuclear factor kappa B (NF-κB) (51). Moreover, it is upregulated during monocyte-macrophage differentiation (52) and tumor necrosis factor (TNF)-α, interleukins (IL)-1β, IL-6, and lipopolysaccharide (LPS) stimulation increases its levels (53). Resistin is also involved in angiogenesis via the induction of endothelial cell growth and migration (54), and can endorse osteoclastogenesis via modulation of bone turnover mediators (51). Thus, bone remodeling and resistin-stimulated pro-inflammatory cytokines in cartilage may promote OA pathogenesis. Nevertheless, further studies are necessary to determine the role of resistin in the onset and progression *in vivo* of OA (40).

Adiponectin, another adipokine produced by adipose tissue, shows higher concentrations within the joint, which suggests an important role in the onset and progression of OA. The synovial fluid of patients with OA shows a 100-fold increase in adiponectin expression as compared with their plasma (55). In addition, adiponectin receptor 1 (AdipoR1) is expressed in cartilage, bone, and synovial tissues (44). Adiponectin may protect against progression of OA since it induces the downregulation of MMP-13 and upregulation of an associated MMP-13 inhibitor (44). Another study suggests that adiponectin may exert an anti-inflammatory effect on cartilage, upregulating the tissue inhibitor of metalloproteinases 2 (TIMP-2) (40, 55). However, serum adiponectin levels are not associated with OA (56).

Experimental models, such as rats, which developed OA after been fed with a high-fat diet (HFD), show that there is a close relationship between obesity and the local immune response in synovial tissue (57). Synovial resident macrophages play a key role in HFD-induced inflammation, which produces an increase in M1 macrophages compared with M2 polarized cells. This highlights the importance of M1 macrophage subsets in the development of obesity-associated OA (57). Furthermore, HFD aggravates synovial inflammation during OA, by increasing macrophage infiltration and metabolic-mediated remodeling of adipose tissue, together with a significant presence of pro-inflammatory factors (58). Additionally, free fatty acids induce activation of Toll-like receptor 2 (TLR2) or TLR4, trigger macrophage recruitment, and activate the NOD-, LRR- and pyrin domain-containing protein 3 (NLRP3) inflammasome (59).

In addition to the secretion of metabolic molecules, obesity contributes to a major mechanical load on the joint. Mechanical loading studies in human and animal models show that abnormal loads can lead to changes in the composition, structure, and mechanical properties of articular cartilage (44), together with inflammatory responses throughout the joint. Mechanoreceptors on the surface of chondrocytes detect increased loading on the knee, triggering intracellular signaling cascades of cytokines, growth factors and MMPs (44).

Osteoarthritic chondrocytes respond differently to mechanical stimulation when compared with cells from healthy joint cartilage (60). The production of catabolic cytokines IL-1β and IL-6, the lack of an increase in mRNA aggrecan levels, and persistent mechanical stimulation are the main differences (61, 62). The mechanotransduction pathway involves recognition of the mechanical stimuli by integrins and activation of integrin-mediated signaling pathways leading to the production of cytokines, in both healthy and osteoarthritic chondrocytes (63).

When we focus on the effect of mechanical loading in OA through the stimulation of pro-inflammatory mediators, *in vitro* mechanical loading experiments show that injurious compression leads to proteoglycan depletion, destruction of the collagen network and cartilage degradation. In response, pro-inflammatory products are released and are postulated to cause synovitis (64). Interestingly, joint movement induces the expression of the anti-inflammatory cytokine IL-10. Therefore, not only the induction of inflammation, but also the lack of resolution of inflammation could play a role in OA (65).

### Type 2 Diabetes and Metainflammation in OA

Type 2 diabetes (T2D) is a metabolic disorder characterized by high blood glucose levels that cause an inadequate β-cell response to the progressive insulin resistance (66). The incidence and prevalence of the most common T2D has nearly doubled in the last two decades, and its presence is reported in a high proportion of knee OA cases (67). Both diseases have many risk factors in common, which may explain the increased prevalence of musculoskeletal diseases in diabetic patients (67).

A considerable association between T2D and OA has been observed in several meta-analyses (68, 69), which is why T2D is the most commonly studied component of MetS as a risk factor for OA (70). Accordingly, epidemiological and experimental evidence suggests that T2D could be an independent risk factor for the onset and progression of OA, that is, the diabetes-induced osteoarthritic phenotype (71, 72). However, other studies show that diabetes mellitus is associated with accelerated degeneration of the cartilage matrix (67) and that T2D is a strong predictor for the development of severe OA, independent of age and body mass index (BMI). This suggests that longstanding diabetes *per se* is detrimental for knee and hip joints (73). According to this, increased levels of the cartilage oligomeric matrix protein (COMP) can be found in the synovial fluid of T2D subjects. Therefore, treating T2D may minimize glycemic control parameters and inflammation, along with synovial fluid COMP levels and OA progression (74).

Furthermore, inflammation plays a key role in the pathogenesis of T2D (59). T2D is associated with low-grade inflammation of adipose tissue in obesity and auto-inflammation in pancreatic islets (75). Indeed, in T2D, pancreas and adipose tissue, as well as other tissues, are infiltrated by macrophages and other immune cells, switching their profiles from an anti-inflammatory to a pro-inflammatory phenotype (76). Pro-inflammatory cytokines inhibit insulin signaling in peripheral tissues and induce β-cell dysfunction, contributing critically to the pathogenesis of T2D (76).

Inflammation in T2D can be triggered by excessive levels of nutrients, like glucose, which leads to the local production and release of cytokines and chemokines: IL-1β, TNF-α, CC-chemokine ligand 2 (CCL2), CCL3 and CXC-chemokine ligand 8 (CXCL8) in the pancreatic islets and insulin-sensitive tissues. In T2D patients, IL-1 receptor antagonist (IL-1RA) production by β-cells is decreased and IL-1β induces inflammation in pancreatic islets through NF-κB activation, which plays a critical role in the development of OA (59). Consequently, immune cells (macrophages and mast cells) are recruited and contribute to tissue inflammation (59, 77) (Figure 2).

Other authors postulate that high levels of glucose stimulate intercellular adhesion molecule 1 (ICAM-1) overexpression in the endothelium. ICAM-1 is a hallmark of diabetes-related inflammation and a crucial driver for cartilage degradation in T2D models. ICAM-1 enables and facilitates the adhesion and entrance of macrophages from serum or the synovium and facilitates the release of cytokines like IL-1β by the chondrocytes or synoviocytes This event triggers the production of MMP-13 and the recruitment of more macrophages, creating a vicious circle (78).

### Senescence and Metainflammation in OA

Aging is a critical risk factor for the onset and progression of many chronic diseases, from metabolic disorders to neurodegenerative diseases, such as Alzheimer's and Parkinson's disease (32, 79). However, the aging process itself does not seem to be the direct cause of these disease states. Aging results from an imbalance between a variety of environmental and cellular stressors, and an insufficient capacity of cellular mechanisms to resolve stress processes. This results in the accumulation of unrepaired damage, which makes individuals more susceptible to developing chronic diseases (80). Also, there is an energy imbalance as a result of an age-associated decrease in the resting metabolic rate, in part due to a loss of lean mass, which does not synthesize enough ATP in older individuals. Finally, there is a dysregulation of signaling cascades that guarantee tissue homeostasis, including hormones, inflammatory mediators and antioxidants, thus triggering an inflammatory state characteristic of the aging process (80) (Figure 1).

This imbalanced situation ultimately leads to decreased adaptation to stress, loss of proteostasis, cellular senescence, inflammatory responses, metabolic disarrangement, DNA damage, detrimental epigenetic modifications, and impaired cell - cell communication. These defective responses trigger a state of low-grade chronic inflammation associated with aging that has been termed “inflammaging,” which plays a major role in the pathogenesis of age-related diseases (32, 79–83).

Aging and age-related diseases share diverse mechanisms that promote low-grade chronic inflammation (32). These mechanisms include inflammatory processes, mainly the chronic activation of innate immunity, but also changes in the adaptive immune system, such as defective T cell functioning, leading to immunosenescence (79). Another prominent mechanism actively contributing to inflammaging is cellular senescence. Senescent cells are unable to proliferate but remain metabolically active and acquire a senescent-associated secretory phenotype (SASP). This SASP is characterized by altered secretion of pro-catabolic enzymes, such as MMP-1, MMP-3, MMP-9, and MMP-13; pro-inflammatory cytokines, such as IL-6, IL-1α and β; chemokines (IL-8, CCL2, CCL5, and CCL19); and the following growth factors: transforming growth factor-β (TGF-β), fibroblast growth factors (FGFs), vascular endothelial growth factor (VEGF) (45, 83). Furthermore, senescent cells exhibit DNA damage, shortening of telomeres, defective autophagy, mitochondrial dysfunction, and increased oxidative stress (32, 41, 83) (Figure 1).

In osteoarthritic cartilage, chondrocytes suffer cellular senescence, known as “chondrosenescence,” as a result of inflammatory signals that increase with aging and mechanical stress (32, 84). An accumulation of senescent cells is also found in the osteoarthritic synovium (41, 83). These factors, together with chondrocyte inability to repair the damaged tissue, are responsible for cartilage degeneration in OA (82). Aging in cartilage is also associated with lower cell density and loss of proteoglycans, joint stiffness, increased chondrocyte size and protein glycosylation (32, 85, 86). Advanced glycation end products (AGEs) increase with age in the cartilage extracellular matrix (ECM) and modify the mechanical properties of cartilage. Their receptor can also interact with TLRs on the surface membrane, thus enhancing innate immune activation (83). Chondrosenescence also occurs through the loss of chondrocyte autophagy. Chondrocytes closely depend on autophagy for the maintenance of cartilage homeostasis, since it is the protective mechanism responsible for the removal of cellular debris and macromolecules through lysosome-mediated degradation (32). In the OA pathogenic context, autophagy reduces inflammation in the joint tissues (87–89). However, the loss of chondrocyte autophagic capacity associated with aging results in further destruction of the cartilage ECM and increased oxidative stress (32, 90). The senescent phenotype in chondrocytes and synoviocytes contributes to perpetuating a local pro-inflammatory state in the joint, due to the release of cytokines and increased cartilage degradation. At the same time, the SASP favors a state of low-grade systemic inflammation through the release of chemokines that also contributes to the pathogenesis of OA (32) (Figure 2).

## Immunity and the Purinergic System in OA

As mentioned above, OA displays low-grade chronic inflammation, primarily mediated by innate immunity (45). Articular cartilage damage and degeneration occurs mainly because of an imbalance between the mechanical load it receives and its ability to absorb and distribute it. Altered mechanical joint loading is often associated with being overweight, anatomical misalignment or post-traumatic joint instability, and these are all critical risk factors for joint destruction (91, 92). The progressive destruction of the cartilage matrix leads to the appearance of tissue fragments in the articular cavity, known as “damage-associated molecular patterns” (DAMPs). These signals are detected by “pattern recognition receptors” (PRRs), present in chondrocytes and synoviocytes. Toll-like receptors (TLRs) are the PRRs that mainly mediate this signaling, particularly TLR2 and TLR4 in OA cartilage (41, 93). TLRs trigger a signaling cascade that results in innate immunity activation, by leading the activation of interferon-regulatory factors, NF-κB, and activator protein 1 (AP-1), and inducing pro-inflammatory mediators, such as cyclooxygenase (COX)-2; cytokines, such as IL-6 and TNF-α; components of the inflammasome, such as caspase-1, NLRP3, pro-IL-1 and pro-IL-18; MMPs and aggrecanases, such as ADAMTS-4 and−5 (41, 45). The activity of MMPs and ADAMTS, enzymes that degrade type II collagen and aggrecan in the cartilage matrix, promote the appearance of more DAMPs, thereby perpetuating tissue damage and innate immunity activation.

Varying extracellular concentrations of different purinergic metabolites, such as ATP, ADP and adenosine, are reliable indicators of the tissue status during inflammation, entailing a robust regulatory system of the immune response (94–97).

Adenosine is a potent modulator of inflammation and immune responses (98). Adenosine regulates function, proliferation and activation of immune cells, and promotes the resolution of inflammation dampening the immune response in physiological and pathological situations, acting as a self-limiting signal (96). Platelets, endothelial cells, neutrophils and macrophages are considered an important source that contributes to increasing extracellular adenosine during inflammation (99) (Table 1). It is well-known that adenosine biases TLR4. Adenosine, by A2AR activation, can inhibit LPS-induced TLR4-mediated responses, by inhibiting Th1-polarizing responses (TNF-α, IL-12p70) and activating anti-inflammatory cytokine production and Th2-polarizing responses (IL-6 and IL-10) (111, 112) and inflammation-resolving properties (113). Adenosine produces increased levels of cAMP, and this second messenger inhibits LPS-induced IL-12 production in murine peritoneal macrophages (114), decreases LPS-induced TNF but enhances IL-10 in human monocytes (115). Nevertheless, the effects of cAMP on TLR-mediated cytokine production can be dependent on cell type. Recent evidence suggests that cAMP levels in chondrocytes are an indicator of the metabolic function, with low levels indicating cartilage degradation, and increased levels suggesting an increased cartilage synthesis (116).

**Table 1:** Effects of adenosine and ATP through their specific purinergic receptors in inflammation.

**Adenosine**			**ATP**	
**Cell type**	**Receptor**	**Effect**	**Receptor**	**Effect**
Endothelial cells	A2AR, A2BR	Blockade of IL-6, IL-8, E-selectin, VCAM-1 (99) Inhibition of the recruitment of leukocytes and neutrophils adhesion to the endothelium (95)	P2	↑ intracellular Ca^2+^, ↑ mitochondria ATP production (100) ↑ NO production (100)
Neutrophils	A2AR	↓ oxidative stress response (99) ↓ leukotriene B4 secretion (99) ↓ IL-8 production (99) ↓ apoptosis (99) ↓ neutrophil-induced NETosis (101)	P2X7	↑ adhesion to endothelial cells (100)
	A2BR	Suppression of neutrophil transmigration across the endothelial vessel wall (102) Inhibition of VEGF secretion (102)	P2Y2, P2Y6, P2Y14	↑ chemotaxis (103) ↑ oxidative stress (104)
	A1R	↑ neutrophil chemotaxis and phagocytosis (105)		
	A3R	Neutrophil migration to injury site (106) ↓ ROS production and chemotaxis (106)		
Macrophages and monocytes	A2AR	Suppression of TNF-α, IL-6, IL-12, IL-8, NO and MIP-1α production (106) ↑ IL-10 production (107) ↑ VEGF, angiogenesis (95, 108)	P2X7	↑ IL-1β production (109) NLRP3 inflammasome activation (109)
	A2BR	↑ IL-10 secretion (95) Inhibition of iNOS and MHC-II expression (99)	P2X4	↑ CXCL5 secretion (110) ↑ ROS production (104) ↑ MIP-2 secretion (104)
	A3R	Regulates macrophage migration (105)	P2Y1, P2Y6, P2X	↑ phagocytosis of apoptotic cells (104)
Dendritic cells	A2AR	Suppression of the capacity to activate naïve T cells (106) Differentiation of T cells into Th1 helper leukocytes (106)	P2X7	Differentiation (100) Secretion of inflammatory cytokines (IL-1β, IL-18, TNF-α, IL-23) (100) Activation of TH-17 lymphocytes (100)
	A2BR	↑ pro-angiogenic activity under hipoxic conditions (105)	P2Y11	↑ cAMP, inhibition of T lymphocyte activation (104) ↓ pro-inflammatory cytokine and chemokine production (104)
	A1R	Loss of the phagocytic capacity (106)		
NK cells	A2AR, A2BR	↓ TNF-α expression (106) ↓ pro-inflammatory cytokine (105)	P2Y11	↓ cytotoxic activity of endothelial CX3CL1 (104)
	A3R	↑ cytotoxic activity (106)		

*↑ means increase; ↓ means decrease*.

As mentioned, TLRs are activated during OA progression, particularly TLR4 (93). Different drugs have been shown to decrease the inflammatory and catabolic response in OA chondrocytes stimulated with different DAMPs by inhibiting the TLR4/MyD88/NF-κB signaling pathway (93). In murine articular chondrocytes, hyaluronan (HA) fragments have been found to induce inflammation via CD44 and TLR4 and NF-kB activation; adenosine can attenuate this inflammation process via A2AR activation (117). Although the exact mechanism has not yet been fully elucidated, it is known that adenosine produces high levels of cAMP that activate protein kinase A (PKA) and inhibit NF-κB (118). No data has been found in the literature, but this mechanism must be altered in OA progression.

In contrast to the anti-inflammatory role of adenosine-mediated purinergic signaling, nucleotide receptors promote inflammatory mechanisms (3). Elevated concentrations of ATP are usually a warning sign of cell death detected by immune cells at sites of active inflammation. Extracellular ATP plays an important role in the innate immune response, upregulating the inflammatory pathways (119). Extracellular ATP (in the millimolar range) predominantly induces proinflammatory effects through activation of the low affinity receptor P2X7 (120), but low (micromolar) extracellular ATP concentrations exert immunosuppressive action through the activation of the high affinity P2Y11 receptor (121, 122).

ATP is considered like a DAMP in OA as it activates TLR4 and leads to NLRP3 inflammasome activation and caspase-1-mediated IL-1β secretion (123) and activates NF-κβ signaling (124), leading to cartilage degradation and synovial inflammation.

## Purinergic System in OA and Modulation by Metainflammation

The direct effect on the joint of purinergic system activation in different cell subtypes has been described extensively (18, 125). Therefore, we shall only concentrate on the modulation of the purinergic system in chondrocytes, since they play a key role in OA.

### Adenosine and Its Receptors in OA

The presence of adenosine receptors in human articular chondrocytes was first discovered in 1999 (126), and they were further characterized in bovine chondrocytes in the presence or absence of low-frequency low-energy pulsed electromagnetic fields (PEMFs) (127). Since then, much importance has been conferred to the role of adenosine in the regulation of inflammatory processes in cartilage and the maintenance of joint homeostasis, modulating the release of pro-inflammatory mediators and cytokines (128). All four adenosine receptors are expressed in chondrocytes, but A2AR and A3R are particularly relevant (18, 22, 128, 129).

A2AR has a broad range of physiological activities, and is known to play an essential role in the maintenance of articular cartilage homeostasis (130). Biophysical interventions, such as PEMFs, for stimulation of bone and cartilage via A2AR are currently being studied (131, 132). There are different modalities of *in vivo* adenosine delivery: polydeoxyribonucleotides, liposomes (129), and functionalized nanoparticles (133). All of them are for intra-articular administration and activate adenosine receptors. When cartilage explants are treated with A2AR antagonists (ZM241385 as a specific antagonist; CGS15943 and theophylline as broad receptor antagonists), cartilage matrix degradation occurs, which is evidenced by increased glycosaminoglycans (GAG), MMP-3, MMP-13, Prostaglandin E2 (PGE2) and NO release (134). It has been observed that in mouse articular chondrocytes stimulated with IL-1β, an A2AR agonist (CGS21680) can counteract the upregulation of the inflammatory markers NF-κB, TNF-α, IL-6, MMP-13, and NO (135). It has also been reported that in the presence of IL-1β, osteoarthritic chondrocytes release less adenosine and ATP, suggesting that inflammation reduces purinergic signaling via A2AR. This may be contradictory to another study which reported that in equine articular chondrocytes, LPS stimulates accumulation of extracellular adenosine working as an inflammatory blocker on chondrocytes (136). The divergence between studies could be due to the nature of the stimuli or species-dependent differences. Mice lacking A2AR develop OA, which can be seen in increase in MMP-13 and Col10a1 expression, fibrillation and thinning of cartilage, disordered chondrocytes, less GAG and loss of sulfated proteoglycans and collagen in cartilage (130). These events are not present in the preventive group or in the treatment group, after intra-articular injection of liposomal adenosine in a rat model of post-traumatic OA (130). Adenosine released by osteoarthritic chondrocytes can also signal via A2AR to limit the production of intracellular NO, which is associated with ECM degradation and chondrocyte apoptosis (129, 137) (Table 2).

**Table 2:** Description of purinergic receptor roles in osteoarthritis (OA), obesity and type 2 diabetes (T2D).

**Purinergic receptors**	**Role in OA**	**Role in obesity**	**Role in T2D**
A1R	It is able to induce analgesia by decreasing nociceptor nerve conduction (138).	A1R activation increases lipogenesis (139, 140), adipogenesis (141) and leptin production (142, 143). Over-expression of A1R in adipose tissue in mice protect from obesity-induced insulin resistance (144). A1R KO mice show increased fat mass and body weight, and impaired glucose tolerance and insulin sensitivity (145, 146).	A1R stimulation induces increased insulin sensitivity and reduces insulin secretion (147).
A2AR	Maintenance of AC homeostasis. Mice lacking A2AR develop spontaneous OA and chondrocytes lacking A2AR develop an OA phenotype (130). Adenosine replacement by intra-articular injection of liposomal suspensions containing adenosine prevents development of OA in rats (130).	A2AR activates lipolysis, induces increased energy expenditure and protects mice from diet-induced obesity (148).	Signaling through the A2AR increases proliferation and survival of β-cells and promotes β-cell regeneration (149, 150).
A2BR	A2BR plays a harmful role in cartilage homeostasis due to its capacity of stimulating inflammatory pathways (128, 151).	A2BR inhibits adipogenesis and lipogenesis and correlates with parameters of obesity. Mice fed with HFD show A2BR upregulated in visceral adipose tissue (152). Obese patients show a positive association between A2BR expression in subcutaneous fat and BMI (149).	A2BR activation increases insulin resistance by affecting the production of IL-6 and other cytokines (153, 154). A2BR affects inflammatory processes in adipose tissue through the activation of macrophages, and indirectly inducing the development of insulin resistance (153).
A3R	It shows an anti-inflammatory effect in OA. KO mice develop progressive loss of AC. OA rat model orally treated with A3R agonist prevented cartilage damage and apoptosis of chondrocytes, osteophyte formation, bone destruction, reduced pannus formation and lymphocyte infiltration (155).	KO animals for this receptor present less abdominal and total body fat (148).	A3R activation induces β-cell necrosis, but the role of this receptor in the regulation of glucose and lipid homeostasis in T2D is unknown (149).
P2X1	At high concentrations of ATP, P2X1 facilitates the release of NO and PGE2, which are involved in inflammatory processes and cartilage resorption (156).	P2X1 is expressed on immune cells and its activation contributes with an inflammatory response. However, its expression during overweight or obesity has not been described (157).	P2X1 is expressed in rodent β-cells, but it cannot be detected in human β-cells (158). T2D does not affect the distribution or the gene expression of P2X1 (159).
P2X2	As P2X1, at high concentrations of ATP, P2X2 facilitates the release of NO and PGE2, which are involved in inflammatory processes and cartilage resorption (156).	Obesity promoted a decrease in the expression of P2X2 receptors on enteric neurons of obese male mice (160).	P2X2 is expressed in rodent β-cells, but it cannot be detected in human β-cells (158). P2X2 expression is decreased in the retina of diabetic rats (161).
P2X3	P2X3 activation in chondrocytes induces NO and PGE2 release, suggesting a role in modulating the inflammatory process and playing an important role in the development of articular hyperalgesia in arthritic joints (162).	-	P2X3 receptors are present in isolated single mouse β-cells, in rat pancreas (158) and in human β-cells (158). P2X3 activation leads to enhanced insulin secretion (158).
P2X4	P2X4 is involved in excessive ATP efflux when ANK gene is overexpressed in OA (163), causing accumulation of crystals (164).	As P2X1, P2X4 is expressed on immune cells and its activation contributes with an inflammatory response. However, its expression during overweight or obesity has not been described (157).	P2X4 is expressed in rodent β-cells, but it cannot be detected in human β-cells (158). At higher extracellular ATP concentrations (more than 1 μM), cell viability decreased and P2X4 is implicated (165).
P2X5	Although it has not been described in OA, it is known that P2X5 signaling contributes to bone loss in experimental periodontitis via promotion of inflammation and direct regulation of osteoclast maturation (166).	P2X5 is present in the surface of brown/beige human adipocytes, with very low expression in white fat. It is also expressed in brown preadipocytes, and its expression is further increased upon differentiation (167).	P2X5 is present in human β-cells, but further investigation is needed to highlight its role (158).
P2X6	–	–	P2X6 is expressed in rodent β-cells, but it cannot be detected in human β-cells (158). Further investigation is needed to highlight its role (158).
P2X7	P2X7 activation in OA chondrocytes aggravates inflammatory process, pain and cell death (168). Overactivation of P2X7 by high concentrations of ATP in OA cartilage areas leads to cell death (74). Blocking P2X7 in rats exert pain-relieving and anti-inflammatory effects (169). P2X7 causes accumulation of crystals (164).	P2X7 shows anti-adipogenic effects (170). P2X7 KO mice present increased body weight and adipocyte hyperplasia in fat pads (171). P2X7 is expressed on immune cells and their activation contributes with anti-inflammatory response. However, its expression during overweight or obesity has not been described (157).	P2X7 is present in β-cells, and it is down-regulated in T2D. In human islets, P2X7 seems to be involved in secretion of insulin (165). The P2X7 KO mice have lower β-cell mass, impaired glucose tolerance and defective insulin and interleukin secretion (172).
P2Y1	Mice P2Y1 KO show reduced trabecular bone in the long bones. Cells derived from mice P2Y1 KO has increased osteoclast formation and resorption (173).	P2Y1 induces adipogenic differentiation of stem cells (170, 174). P2Y1 is able to induce leptin production in murine adipose tissue (175).	P2Y1 is present on intra-islet capillaries and in small pancreatic blood vessels. At higher extracellular ATP concentrations (more than 1 μM), cell viability decreased and P2Y1 is implicated (165).
P2Y2	It is involved in the signaling of mechanical forces coming from the extracellular matrix articular via integrins. OA chondrocytes do not increase the amount of extracellular ATP via P2Y2 after mechanical stimulation, and do not stimulate anabolic responses (63, 176).	P2Y2 induces the adipogenic differentiation of stem cells (170, 174). Mice with depletion of P2Y2 show dependant resistance to develop HFD-induced obesity accompanied with an improvement of the metabolic status (177).	P2Y2 is expressed in small pancreatic blood vessels, but T2D does not affect the distribution or the gene expression of P2Y2 (159).
P2Y4	In the periodontal ligament, P2Y4 induces phosphorylation of ERK and consequently type I collagen and OPG release, essential for remodeling of the alveolar bone. However, its role in OA has not been describe yet (18).	P2Y4 is able to induce the adipogenic differentiation of stem cells (170, 174). Activation of P2Y4 inhibits adiponectin expression, and P2Y4 KO mice show increased adiponectin secretion (178).	P2Y4 has been identified in pancreatic duct cells of the young rat (α and β-cells), but its role in T2D must be clarified (179).
P2Y6	As P2Y4, in the periodontal ligament P2Y6 induces phosphorylation of ERK and consequently type I collagen and OPG release, essential for remodeling of the alveolar bone. But its role in OA has not been describe (18).	Selective P2Y6 deficiency in mice AgRP neurons prevents diet-induced hyperphagia, adiposity, and insulin resistance in the long term (180). P2Y6 deficiency blunts macrophage-inflammatory responses and limits atherosclerosis development (180).	Mouse islets cells possess P2Y6 receptor, whose activation lead to the modulation of insulin secretion. It may play a role as autocrine regulator of insulin release (181).
P2Y11	–	P2Y11 induces the adipogenic differentiation of stem cells (170, 174), but further research is necessary to understand its role in obesity.	In human and murine tissue there is evidence that P2Y11 is a key upstream component in the signaling cascade regulating vascular reactivity during diabetic hyperglycemia (182).
P2Y13	–	P2Y13 shows anti-adipogenic effects (170). Mice P2Y13 KO improve outcome in metabolic syndrome with an increased protection against developing an insulin resistance as shown through an improved glucose tolerance and basal glucose levels, a decelerated weight gain and a better metabolic turnover (183).	P2Y13 is involved in the induction of β-cell apoptosis in presence of high glucose and free fatty acids levels (184).
P2Y14	–	P2Y14 shows anti-adipogenic effects (170).	P2Y14 deficiency mice significantly changed expression of components involved in insulin secretion.

*AC, Articular cartilage*.

A3R exerts anti-inflammatory effects on different experimental OA models (185). A3R knockout (KO) mice develop progressive loss of articular cartilage. Agonists for A3R downregulate key genes implicated in OA pathology, such as RUNX2 (151). In a recent study CF101, a highly selective A3R agonist, was orally administered twice daily to monosodium iodoacetate OA-induced rats. It was found that CF101 downregulated the signaling pathway of NF-κB, which led to decreased levels of TNF-α. This effect prevented cartilage damage and chondrocyte apoptosis, osteophyte formation, and bone destruction. It also reduced pannus formation and lymphocyte infiltration. On the other hand, all these effects were counteracted by the A3R antagonist MRS1220 (155) (Table 2).

During inflammatory processes, ADA is secreted to the extracellular space altering adenosine levels. Synovial fluid ADA measurement, in association with C-reactive protein (CRP) and erythrocyte sedimentation rate (ESR) levels, makes it possible to distinguish OA from other rheumatic diseases, like RA (186). In cartilage, when extracellular adenosine levels decrease, cartilage damage markers appear, e.g., increase in GAG release, and higher production of MMPs, PGE2 or NO (187). In some rheumatic diseases like RA, ADA1 levels are increased, compromising extracellular adenosine levels and possibly contributing to the severity of the disease (188).

Pain intensity correlates with OA progression. At low concentrations, adenosine binds to A1R inducing analgesia by decreasing nociceptor nerve conduction, and to A2AR, which triggers an anti-inflammatory response by secreting anti-inflammatory mediators. However, excessive adenosine concentration can be detrimental (138). In the case of excessive joint motion, which leads to excessive activation of CD73 via hypoxia inducible factor (HIF)-1α, extracellular adenosine accumulates. The consequent activation of the low-affinity A2BR seems to play a harmful role in cartilage homeostasis, possibly due to its capacity of stimulating inflammatory pathways involving MAPK (128, 151). In OA, as a low-grade chronic inflammatory disease, increased extracellular adenosine levels may switch selectivity of receptor binding toward A2BR, generating hyperalgesia (138) (Table 2).

As mentioned above, inflammation reduces signaling via A2AR in chondrocytes. However, in response to metabolic stress and inflammation, adenosine accumulates extracellularly, like in obesity (189). The activation of the adenosine A1R increases lipogenesis (139, 140), adipogenesis (141) and leptin production (142, 143). Accordingly, overexpression of A1R in adipose tissue in mice protects from obesity-induced insulin resistance (144), whereas A1R KO mice show anti-lipolytic effects (139). A1R KO mice exhibit increased fat mass and body weight, and impaired glucose tolerance and insulin sensitivity (145, 146). Contrary to A1R, the activation of A2BR, which is highly expressed in human primary pre-adipocytes (148), can inhibit both adipogenesis and lipogenesis *in vitro* (141, 190). Moreover, in rodent models, the overexpression of A2BR seems to correlate with parameters of obesity, being upregulated in visceral adipose tissue of mice fed HFD (152). Finally, studies in obese patients show that A2BR expression in subcutaneous fat is positively associated with BMI and other parameters of obesity (149). On the other hand, activation of A2AR in human and murine adipose tissue not only activates lipolysis, but also induces increased energy expenditure and protects mice from diet-induced obesity (148). Finally, the receptor A3R is also expressed in adipocytes, and KO animals for this receptor present less abdominal and total body fat (148) (Table 2).

Furthermore, obesity is closely related to a state of insulin resistance, which is considered to be a key step in the development of diabetes and MetS (190). The stimulation of A1R induces insulin sensitivity and reduces insulin secretion (147). On the other hand, the activation of A2BR contributes to increased insulin resistance by affecting the production of IL-6 and other cytokines (153). Animal studies confirm that A2BR activation increases serum IL-6 levels (154), which may be involved in the development of insulin resistance and improve insulin sensitivity (191). A2BR affects inflammatory processes in adipose tissue through the activation of macrophages, and indirectly inducing the development of insulin resistance (153) (Table 2).

In view of the above, these changes in the purinergic system mediated by MetS could be involved in the development of OA, which would partially explain the relationship between both pathologies. It has been stated that in adipose tissue there is a low stimulation of A1R and A2AR in obesity conditions (148). Interestingly, this situation is similar in chondrocytes, which could induce a loss in cartilage homeostasis, since they play an essential role in the maintenance of tissue, and consequently promote the development of OA (130). Moreover, the activation of A2BR in obesity contributes to inflammatory processes and secretion of pro-inflammatory molecules (148), which possibly contribute to the cartilage alteration seen in OA. According to this, new studies are being developed and show that intra-articular stimulation of A2AR can reverse not only OA induced by anterior cruciate ligament injury, but also obesity-related OA in experimental models of the disease (192).

As described above, T2D is the most commonly studied component of MetS as a risk factor for OA. Metformin, a first-line drug for T2D treatment, is known to affect the energy state of the cell. It has been recently proposed that metformin may be beneficial in obese patients with knee OA (193) and can also inhibit respiratory chain complex 1, activating AMPK and inhibiting AMP deaminase, which results in an increase in extracellular adenosine (194). The increased adenosine levels might activate A2AR and could explain the beneficial effect of metformin in obese patients with knee osteoarthritis, but this needs to be explored further.

### P2 Receptor Modulation in OA

Currently, it is known that extracellular nucleotides are fundamental in the regulation of biological processes in many tissues, including the musculoskeletal system, and could work as potential therapeutic targets (195).

We have known that nucleotide receptors are expressed by chondrocytes and are associated with PGE2 release since 1991 (196). However, their role in chondrocytes may be controversial, possibly depending on the amount of extracellular ATP available and the physiological/pathological conditions of the joint (18). Regarding the physiological conditions, prechondrogenic condensation occurs, a phenomenon necessary for chondrogenic differentiation and skeletal patterning. This process of prechondrogenic condensation is mediated by extracellular ATP signaling via the P2X4 receptor, which leads to Ca^2+^-driven ATP oscillations, ensuring the correct constitution of the joint (197). P2X1 and P2X2 receptors were identified in primary bovine chondrocytes, showing that they can facilitate the release of NO and PGE2, which are involved in inflammatory processes and cartilage resorption, in the presence of high concentrations of ATP (156). Upregulation of proteoglycan levels and reduction in NO release after dynamic compression occurs in bovine articular chondrocytes via P2 and has an anabolic effect on the cartilage (198). P2X2 and P2X3 receptors are also expressed in chondrocytes. ATP levels in OA have been linked to pain intensity (199). Activation of P2X3 receptors in chondrocytes induces NO and PGE2 release, suggesting their function in modulating the inflammatory process and playing an important role in the development of articular hyperalgesia in osteoarthritic joints (162) (Table 2).

The P2X7 receptor is highly expressed in cells of the immune system, and participates in regulating inflammation and pain, although the exact mechanisms are not yet understood (200). Interestingly, the P2X7 receptor needs a high concentration of ATP for full activation, suggesting a specific role under pathological conditions (201). Accumulation of ATP released by osteoarthritic chondrocytes could act as a warning signal via P2X7, aggravating the inflammatory process and pain and causing cell death (168). Low concentrations of extracellular ATP produce a positive response of the P2X7 receptor activation toward cell proliferation. However, when the concentration of ATP is high, as it is supposed to be in cartilage areas affected by OA, there is an overactivation of the P2X7 receptor, leading to cell death (47). The strategy of blocking the P2X7 receptor could benefit the survival of chondrocytes in OA. It has been found that signaling via P2X7 in rabbit articular chondrocytes leads to chondrocyte apoptosis through the activation of the phospholipase A2 (PLA2)/cyclooxygenase-2 (COX-2) pathway (45). Numerous studies have focused on pain mitigation, one of the main consequences of OA, and their results show that targeting purinergic receptors may be considered as a therapeutic alternative to stop or slow down articular cartilage degeneration. Blocking the P2X7 receptor would have an impact on cartilage itself since it has been observed that its inhibition by the selective antagonist AZD9056 exerts pain-relieving and anti-inflammatory effects. This antagonist counteracts the induction of MMP-13 or NF-κB, both upregulated in osteoarthritic chondrocytes, in an OA rat model. In this study, AZD9056 also reversed the upregulation of IL-1β, IL-6, TNF-α, substance P and PGE2 (169) (Table 2).

Extracellular inorganic pyrophosphate, whose levels regulate physiological and pathological mineralization, are also influenced by P2 receptors. Suramin, a putative antagonist of P2 receptors, can block the stimulation generated by ADP on the production of inorganic pyrophosphate in chondrocytes, with the consequent blockade of excessive deposition of calcium pyrophosphate dihydrate (CPPD) in the articular cartilage, which would hinder the correct mineralization of the bone (202). Similarly, and consolidating the idea that high concentrations of extracellular ATP promote cartilage damage, another study on primary articular chondrocytes has shown that P2X7 and P2X4 may be implicated in excessive ATP efflux when the ANK gene is overexpressed, as in OA (163), causing accumulation of CPPD (164). Modulation of these receptors would facilitate the reduction of joint damage and cartilage regeneration (Table 2).

However, activating or blocking P2 receptors in OA does not only act on the inflammatory onset of the disease. As mentioned above, abnormal mechanical loading of the joint exerts changes in cell structure and mechanical properties that lead to OA. Physiological joint loading and articular cartilage compression seem to play a key role in ATP release and P2 signaling. Mechanical forces coming from the ECM are transmitted to rat articular chondrocytes via integrins, activating different molecular mechanisms, such as stimulation of the P2Y pathway, mainly by P2Y1, P2Y2, and P2Y4 receptors (203). A candidate to transfer these forces is the primary cilium, which modulates ATP-induced Ca^2+^ signaling via P2X (P2X4, P2X7) and P2Y (P2Y1, P2Y2) receptors (204). As an overview, the forces applied to chondrocytes lead to an increase in ATP release, which downregulates the expression of MMPs, such as MMP-13 (205). On the contrary, when sustained pressure is maintained on chondrocytes, ATP release is suppressed, causing cartilage degeneration (206). This mechanical stimulation is essential for maintaining cartilage integrity, and different responses to this input have been observed between healthy and osteoarthritic chondrocytes. Healthy chondrocytes increase the amount of extracellular ATP after mechanical stimulation via the P2Y2 receptor exerting anabolic responses, while osteoarthritic chondrocytes do not increase these ATP levels (63, 176) (Table 2).

From another perspective, the activation of some receptors, such as P2Y1, P2Y2, P2Y4, and P2Y11, induces the adipogenic differentiation of stem cells (170, 174), whereas, other receptors, like P2Y13, P2Y14, and P2X7 show anti-adipogenic effects (170). In addition, the P2Y1 receptor can induce leptin production in murine adipose tissue (175). On the other hand, activation of the P2Y4 receptor inhibits adiponectin expression, and P2Y4 KO mice show increased adiponectin secretion (178). P2X7 KO mice have increased body weight and adipocyte hyperplasia in fat pads (171) (Table 2).

Rat models have revealed that the P2Y1 receptor is present on intra-islet capillaries, and P2X4 receptors appear in β-cells. Moreover, P2X1, P2X3, P2Y1, and P2Y2 receptors are expressed in small pancreatic blood vessels, and β-cells present P2X7 receptors, which are downregulated in T2D. In human islets, the receptor seems to be involved in the secretion of insulin and IL-1RA (165). The P2X7 KO mice show lower β-cell mass, impaired glucose tolerance, and defective insulin and interleukin secretion (172). Furthermore, the P2Y13 receptor is involved in the induction of β-cell apoptosis in the presence of high glucose and free fatty acid levels (184). Extracellular ATP (1 μM) increases insulin secretion in mouse β-cell lines, but at higher ATP concentrations, cell viability decreases, with involvement of the P2Y1 and P2X4 receptors (165) (Table 2).

There is some controversy in the modulation of these receptors both in OA and MetS. Extracellular concentrations of ATP modulate the activation of these receptors, which act differently according to the tissue where they are expressed. The P2X7 receptor is related to the inflammatory processes during OA development, with high levels of extracellular ATP (168). However, in adipose tissue, this receptor shows an anti-adipogenic effect (171). In T2D, the P2X7 receptor has been reported to be decreased (172). Furthermore, activation of the P2Y1 receptor increases in obesity in the adipose tissue (175) and in T2D in β-cells (165). In chondrocytes, the P2Y1 receptor plays a key role in the response to mechanical forces, and when the pressure is maintained, increased ATP release may cause cartilage degeneration (203). A deeper knowledge of these receptors in these three conditions is necessary to better describe the close relationship among them (Table 2).

### Mitochondrial Metabolism, Purinergic Signaling, and OA

**Figure 3:**
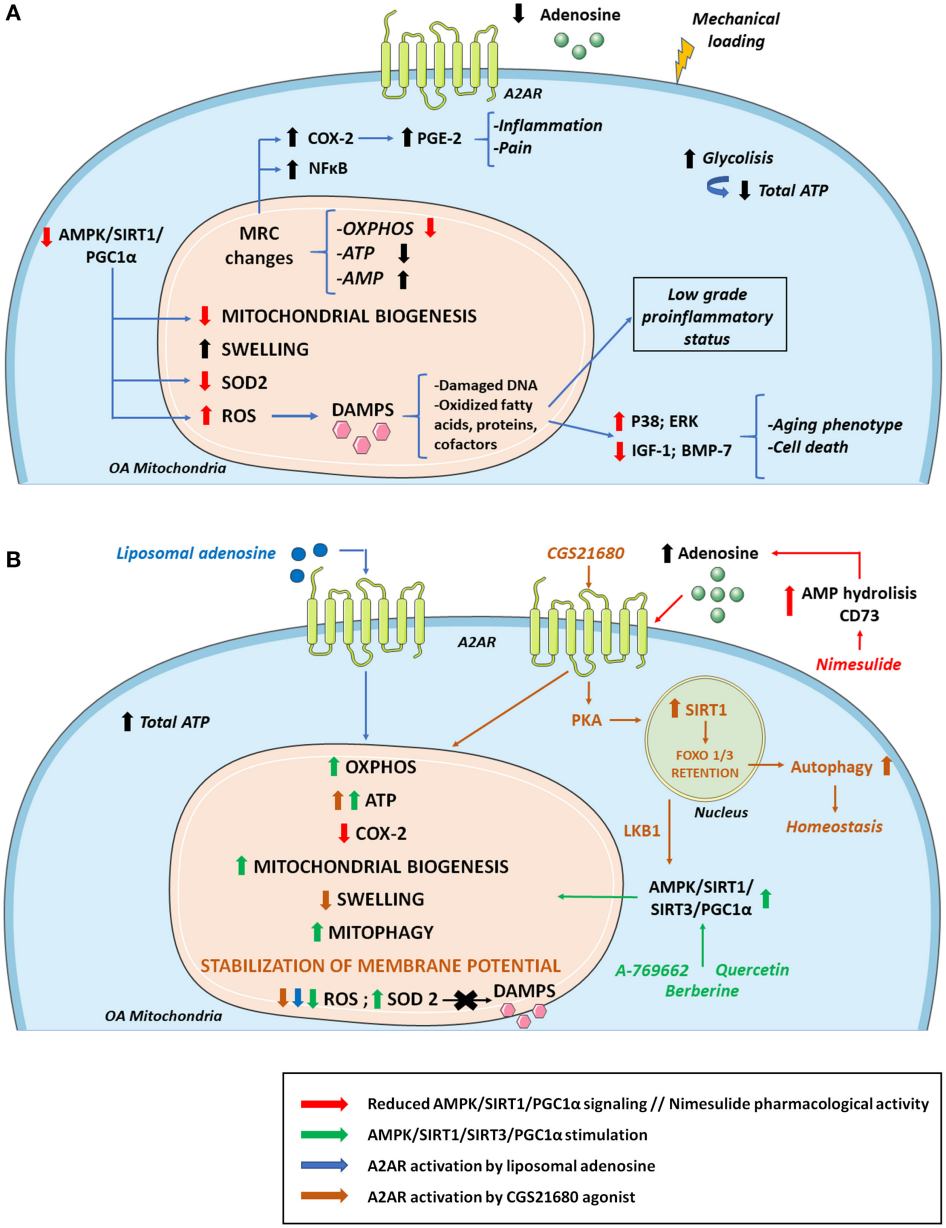
Osteoarthritis (OA) mitochondrial metabolism and its modulation by A2AR and AMPK stimulation. **(A)** During the development of OA, cellular metabolic alterations occur. There is an increase in glycolysis activity to compensate the energy requirement necessary to repair damaged cartilage. However, total ATP levels decrease due to mitochondrial defects together with sustained mechanical loading. Changes in the MRC and its complexes impair ATP and AMP production. As a result, there are lower extracellular levels of ATP and adenosine which diminish A2AR signaling. These alterations in the MRC cause an increase in proinflammatory molecules and transcription factors such as COX-2 and NF-κB, respectively, aggravating inflammation and pain. Reduced AMPK/SIRT1/PGC-1α signaling compromises mitochondrial biogenesis and OXPHOS. There is also a decrease in antioxidant enzymes such as SOD2 that cause increases in ROS production, contributing to the release of DAMPS that aggravate the inflammatory status, stimulating catabolic pathways (p38; ERK) and reducing anabolic pathways (IGF-1; BMP-7), which supposes cell death and an aging phenotype. **(B)** Mitochondrial OA impairment can be partly reversed by AMPK and A2AR stimulation. Quercetin, berberine and pharmacological AMPK activator A-769662 can activate AMPK/SIRT1/PGC-1α and AMPK/SIRT3 pathways increasing OXPHOS, ATP, mitochondrial biogenesis, mitophagy, and SOD2 activity. AMPK stimulation also promotes a decrease in ROS production with reduced DAMPs secretion diminishing inflammation and aging phenotype. Regarding ROS level reduction, similar effects are found when A2AR is activated by agonists like liposomal adenosine. Moreover, A2AR agonist CGS21680 stimulates SIRT1 via PKA allowing FOXO1/3 retention and increased autophagy. CGS2168 indirectly activates AMPK through LKB1. Nimesulide increases extracellular adenosine levels favoring a reduction in the expression of COX-2 and the associated inflammation and pain. AMP, adenosine monophosphate; AMPK, AMP activated protein kinase; BMP-7, bone morphogenetic protein 7; DAMPS, damage associated molecular patterns; IGF-1, insulin growth factor 1; LKB1, liver kinase B1; MRC, mitochondrial respiratory chain; OXPHOS, oxidative phosphorylation; PGC-1α, peroxisome proliferator-activated receptor-γ coactivator-1α; PKA, protein kinase A; SIRT1, sirtuin 1; SIRT3, sirtuin 3; SOD2, superoxide dismutase.

During chondrosenescence, the glycolysis pathway is overstimulated, trying to generate ATP promptly, which is necessary for repairing damaged cartilage (207). Chondrocytes are mainly glycolysis-dependent cells, but they keep the ability to use mitochondrial respiration in certain cases to enhance cell survival and ECM biosynthesis in periods of nutrient stress to sustain ATP synthesis (208, 209). However, under OA conditions, mitochondrial biogenesis and activity are disrupted, leading to a greater amount of ROS production. This can be linked to AMPK deficiency in OA, a molecule responsible for regulating cellular metabolism and energy balance (210). AMPK is also a chondroprotective molecule that is capable of inhibiting procatabolic responses to inflammation and biomechanical injury via its downstream targets, peroxisome proliferator-activated receptor gamma coactivator (PGC)-1α and forkhead box O (FOXO)3A (211). AMPK/sirtuin (SIRT)-3 signaling protects mitochondria from oxidative stress by deacetylating superoxide dismutase (SOD2) and decreasing ROS. This pathway also prevents mitochondrial DNA damage by activating mitophagy (212). Some approaches have focused on enhancing the AMPK/SIRT1 pathway, treating an OA rat model with quercetin, and finding overall improvement in mitochondrial function, higher ATP levels in mitochondria, increased mitochondrial copy number and attenuation of ROS levels (213). Other studies have observed that pharmacological stimulation of AMPK increases PGC-1α via SIRT1, reversing impairments in mitochondrial biogenesis, oxidative phosphorylation (OXPHOS) and intracellular ATP in human knee OA chondrocytes (214). It is established that extracellular adenosine can contribute to AMPK activation and regulation (215). Adiposomal injections and CGS21680 A2AR agonist have demonstrated FOXO1/3 activation and retention in the nucleus, which is implicated in increased autophagy and cartilage homeostasis. This recent study provides a mechanism in which A2AR can activate SIRT1 and AMPK through PKA (216) (Figure 3).

Age-associated mitochondrial dysfunction and mitochondrial dysfunction in OA contributes to perpetuating the low-grade systemic pro-inflammatory status by further promoting cellular senescence and SASP, and with it, the pathological processes associated with inflammaging (32, 210, 217). In this context, mitochondria increase the production of superoxide anion and hydrogen peroxide, and the amount of AMP increases with respect to ATP, and mitochondrial danger-associated molecular patterns (DAMPs) are released. The increase in ROS, DNA damage, oxidized fatty acids, proteins, and cofactors, ultimately alters cellular function (32). This chondrocyte ROS increase is partly due to mitochondrial dysfunction and reduced SOD2 activity, which is an antioxidant enzyme and a by-product of the mitochondrial electron transport chain. The altered redox environment promote disruptions in signaling, increasing pro-catabolic MAP kinases p38 and ERK and inhibition of anabolic insulin growth factor 1 (IGF-1) or bone morphogenic protein 7 (BMP-7) signaling, leading to an aging phenotype and chondrocyte death (218). In OA, this increase in oxidative stress also disrupts proteoglycan and collagen network, favoring the appearance of residues that further activate innate immunity. The accumulation of ROS accelerates cellular senescence processes and apoptosis by decreasing ATP levels (32). Sustained mechanical loading is essential in OA for reducing ATP levels and respiratory activity, and for increasing ROS production on bovine osteochondral explants (206) (Figure 3).

Inflammation and mitochondrial disruption are closely related and affect OA progression. Inhibition of the mitochondrial respiratory chain induces inflammatory responses. In primary human chondrocytes the inhibition of complexes III and V induces COX-2 expression and PGE2 production, the generation of ROS and the activation of NF-κB (219). Therefore, COX-2 levels are upregulated in inflamed joint tissues and are located in the superficial layers of human cartilage with OA, where damage firstly appears (219). COX-2 overexpression is related to higher PGE2 production in osteoarthritic cartilage, which is partly responsible for inflammation and pain (220). Nimesulide, a COX-2 inhibitor, mediates its anti-inflammatory effects *in vivo* and *in vitro* by increasing CD73 activity and AMP hydrolysis so that higher levels of adenosine are available for A2AR activation (221). This mechanism of action positions adenosine signaling as a major player in inflammatory-derived pain mitigation by non-steroidal anti-inflammatory drugs (NSAIDs) and points out an correlation between mitochondrial alterations, inflammation and pain.

Osteoarthritic chondrocytes exhibit a depletion in the mitochondrial production of ATP with a consequent reduction of extracellular adenosine and A2AR stimulation, disrupting chondrocyte homeostasis (222). This ATP level decrease in OA contributes to an increase in glycolysis with pyruvate conversion to lactate and defects in OXPHOS, leading to mitochondrial dysfunction (209). A2AR-null mice develop spontaneous OA with increased mitochondrial dysfunction, increased ROS burden and reduced ATP production via OXPHOS (22, 130). This model also shows mitochondrial depolarization, swelling, fragmentation and mitophagy (222). ROS burden is mitigated in a mouse model of obesity-induced OA by intra-articular injections of liposomal adenosine or CGS21680. This A2AR agonist also increases mitochondrial basal rates of respiration and ATP production *in vitro*, with increased ATP release in IL-1β-treated human chondrocytic T/C-28a2 cells. A2AR activation also stabilizes mitochondrial membrane potential and reduces mitochondrial swelling after IL-1β exposure (222) (Figure 3).

### Purinergic System, Inflammasome and OA

The NLRP3 inflammasome is considered an important component involved in OA pathogenesis (223). Inflammasomes are multi-protein complexes mainly present in the cytosol of myeloid cells, such as neutrophils, monocytes and DCs, but they are also found in non-hematopoietic lineage cells such as endothelial fibroblasts or chondrocytes (224).

Assembly and activation of the inflammasome components by different stimuli (e.g., DAMPs) triggers a regulated pro-inflammatory reaction and induces cellular pyroptosis as a protection mechanism orchestrated by innate immunity to restore tissue homeostasis (225). In the joint, inflammasome dysregulation can lead to synovial inflammation and cartilage destruction, characteristic of OA pathogenesis (226).

**Figure 4:**
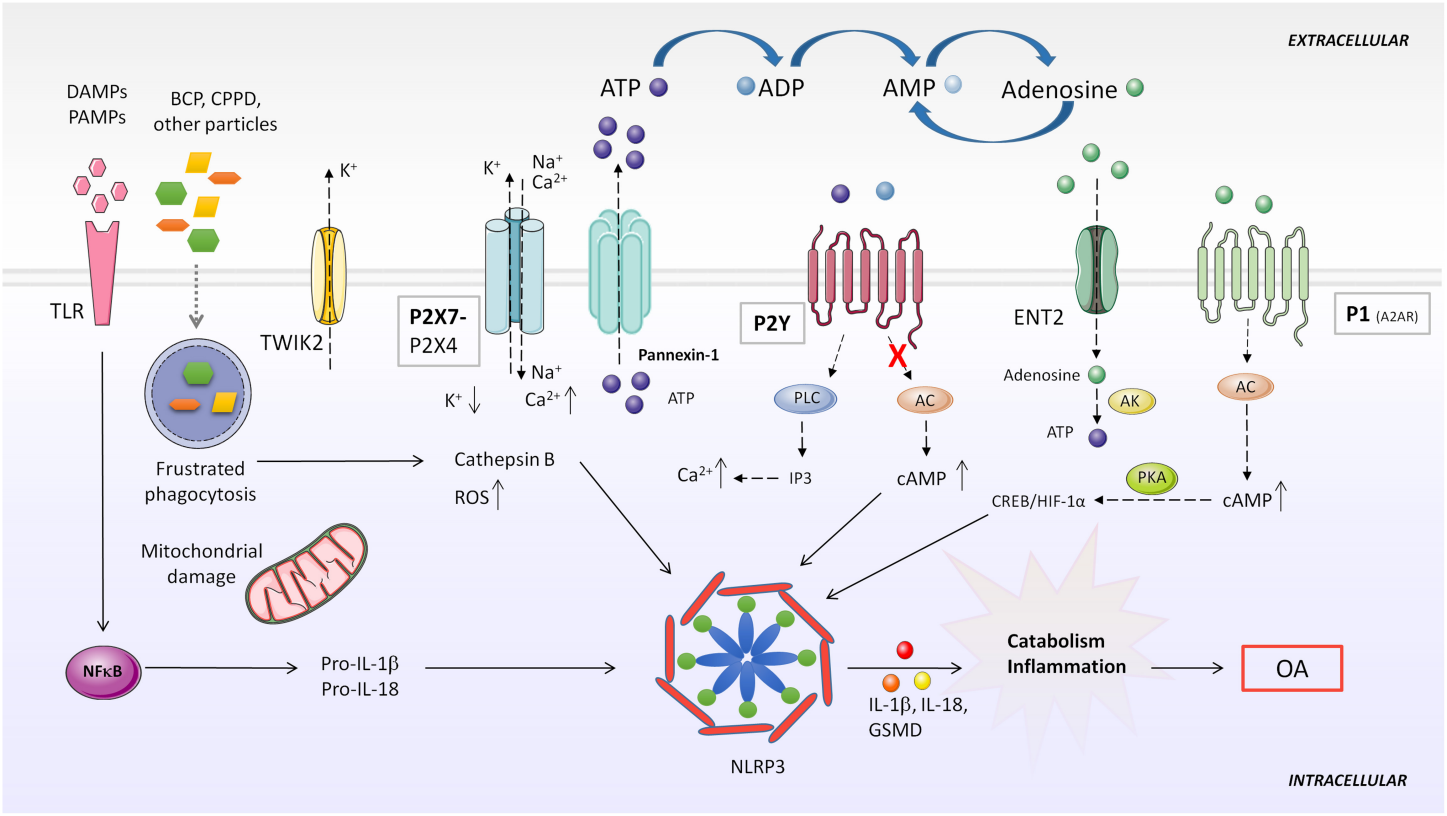
Purinergic system implication in NLRP3 inflammasome activation. DAMPS or PAMPs present in the microenvironment are recognized by TLR receptors, which in turn, upregulate pro-IL-1β and pro-IL-18 expression through NF-κB activity. BCP and CPPD crystals can be both recognized by TLRs and phagocytized by the cell. Impaired particle or crystal phagocytosis by phagosomes leads to cathepsin B release and mitochondrial dysfunction, which induce elevated ROS production and consequent NLRP3 activation. Additionally, frustrated phagocytosis can lead to massive ATP release through Pannexin-1, activatingP2X7 and causing ionic cytoplasmic imbalance. Excessive K^+^ efflux, especially through TWIK2, and Na^+^ and Ca^2+^ influx through P2X7 activates NLRP3 inflammasome. Activation of different P2Y by ATP or ADP increases intracellular Ca^2+^ concentration. Particles can also impede induction of purinergic AC-cAMP pathway allowing NLRP3 activity. A2AR activation by extracellular adenosine induces HIF-1α upregulation, which activates NLRP3. AC, adenylate cyclase; AK, adenosine kinase; BCP, basic calcium phosphate; CPPD, calcium pyrophosphate dehydrate; DAMP, danger-associated molecular patterns; ENT2, equilibrative nucleoside transporter 2; GSMD, gasdermin D; IP3, inositol 1,4,5-trisphosphate; NLRP3, NOD-like receptor pyrin domain containing 3; PAMP, pathogen-associated molecular patterns; PKA, protein kinase A; PLC, phospholipase C; ROS, reactive oxygen species; TLR, toll like receptor; TWIK2, two-pore domain weak inwardly rectifying K^+^ channel 2.

It has been established that at least two signals are necessary to induce NLRP3 activation: a first priming signal, where TLRs recognize DAMPs or PAMPs promoting synthesis of inflammasome components mediated through NF-kB; and a second activation step, which leads to the assembly of the scaffold inflammasome proteins and recruitment of pro-caspase-1. This second signal is stimulated by cellular stress or pathogen-derived molecules (227) (Figure 4).

Although the exact role of NLRP3 is still controversial, several studies have pointed out that overactivation of the NLRP3 inflammasome is involved in low-grade chronic inflammation during OA development (226, 228). Inflammasome secretion of pro-inflammatory IL-1β leads to synovial inflammation and cartilage degradation (229). IL-18, another cytokine processed by the NLRP3 inflammasome, is elevated in synovial fluid and serum of patients with OA, and has been proven to decrease type II collagen and aggrecan expression, disrupt autophagy and induce apoptosis in chondrocytes (230, 231). A recent work shows that inhibition of the mTOR pathway after IL-18 treatment has protective effects in chondrocytes both *in vitro* and *in vivo* (232). Data suggest that IL-1β and IL-18 are mainly produced in the damaged synovium rather than cartilage (233). Analyses of knee joint explants from patients with primary OA have demonstrated that the synovium expresses high levels of NLRP3 inflammasome components (234), and NLRP3 and NLRP1 inflammasomes are overexpressed in synovium derived from knee OA patients (235). In addition, oxidative stress-mediated NLRP3 inflammasome activation was enhanced after inhibiting HO-1 expression (236). Pyroptosis mediated by NLRP3 inflammasome have also been found in chondrocytes *in vitro* and *in vivo* (234).

All these data support the idea that the NLRP3 inflammasome is involved in the onset and progression of OA and can even be considered a potential new biomarker for diagnosis and tracking in patients with OA (228).

Usually, CPPD and basic calcium phosphate (BCP) crystal deposits are found in the synovial fluid and cartilage of patients with OA. Both crystals are believed to be involved in joint inflammation and may correlate with disease harshness (237, 238). BCP crystals can promote NLRP3 inflammasome-dependent or -independent inflammation (239). Although some reports challenge this assumption, like one performed in a mouse meniscectomy OA model, overwhelming data support the notion that BCP and CPPD crystals induce synovial inflammation (240). *In vivo* and *in vitro* models have shown that these crystals are recognized by innate immune system cells, activating the NLRP3 inflammasome (237, 241). TLRs can recognize CPPD and BCP crystals, and their phagocytosis may induce ATP release, K^+^ efflux and ROS production (242), suggesting that the purinergic system plays an important role in crystal-mediated inflammasome activation processes in OA.

ATP, its derived metabolites and their recognition by P2X7, P2Y or adenosine receptors are a fine-tuned regulatory mechanism of inflammasome activity, sustaining a chronic inflammatory condition in different diseases. However, their exact role during OA development remains uncertain. Below we address different mechanisms of inflammasome activation mediated by purinergic signaling. This may open a new insight for understanding OA pathogenesis.

### Adenosine, Its Receptors and the Inflammasome

Extracellular adenosine activates inflammasome-mediated IL-1β production through A2AR, A2BR, and A3R (243). Adenosine influx through nucleoside transporter ENT2 was converted to ATP by AK, increasing intracellular ATP concentration, which led to ATP secretion from macrophages and consequent upregulation of NLRP3 and IL-1β secretion. On the other hand, ADA reduced IL-1β secretion mediated by nanoparticle-induced NLRP3 inflammasome activation in macrophages (243). Another paper demonstrated that after LPS or ATP stimulation of mouse peritoneal macrophages, adenosine prolonged inflammasome activation, upregulating NLRP3 and IL-1β and enhancing caspase-1 activity. Adenosine stimulated A2AR, activating the downstream cAMP/PKA and CREB/HIF-1α pathway (Figure 4). In this study, adenosine was able to overcome a tolerant unresponsive state acquired by macrophages after previous LPS stimulation, re-inducing high levels of IL-1β secretion (244).

### ATP, Its Receptors and the Inflammasome

In normal conditions, excessive extracellular ATP is degraded by ecto-ATPases to adenosine. However, when homeostasis is disrupted, high levels of extracellular ATP released to the milieu from stimulated or necrotic cells acts as a warning signal, being detected by the P2X7 receptor. This results in a massive efflux of K^+^ across the porous cytoplasmic membrane (245). The P2X7 receptor mediates NLRP3 inflammasome activation in different inflammatory diseases and conditions, and is highly expressed in leukocytes and in other cell types (246–248). Pannexin-1 forms hemichannels across the plasma membrane permeable to ATP, releasing it to the extracellular milieu (249). Pannexin-1 directly interacts with the P2X7 receptor in different cell lines, and this has been associated with NLRP3 inflammasome activation (249–251) (Figure 4).

Riteau et al. demonstrated that several sterile particles induced endogenous ATP release from human macrophages in a P2X7 receptor-dependent way. They proposed that ATP release through Pannexin-/Connexin hemichannel activation could be a consequence of cathepsin B leakage from lysosome disruption. Extracellular ATP could then act in an autocrine/paracrine way, activating the P2X7 receptor and amplifying ATP release and inflammasome activation response (252) (Figure 4).

An alternative mechanism was described by Di et al. through which the P2X7 receptor could mediate K^+^ efflux in macrophages. The two-pore domain weak inwardly rectifying K^+^ channel 2 (TWIK2) is involved in NLRP3 inflammasome activation in an LPS-induced lung inflammation mouse model. They proposed that ATP activated P2X7 receptor-favored influx of Na^+^ and Ca^2+^ cations, modulating membrane potential and promoting efflux of K^+^ through the TWIK2 channel (253).

ROS are considered another well-known stimulus through which the NLRP3 inflammasome can be activated. It has been reported that Ca^2+^ influx mediated by the activated P2X7 receptor can phosphorylate NADPH oxidase complexes in different cell types, including macrophages, stimulating the production of ROS (246) (Figure 4).

Although the P2X7 receptor has been described as the main purinergic receptor involved in NLRP3 inflammasome activation, a recent study in rat urothelial cells showed that treating cells with both P2X7 and P2X4 inhibitors eliminated intracellular caspase-1 activity, suggesting the involvement of the P2X4 receptor in inflammasome activity (254).

Riteau et al. also observed that at low ATP doses, the addition of the metabolites ADP and UTP, which act specifically on P2Y receptors, enhanced particle-mediated IL-1β production (252). Oxidized ATP, an inhibitor of P2X and P2Y receptors, was able to decrease IL-1β production in a dose-dependent manner, but P2X7 receptor selective inhibition did not impair IL-1β production. Interestingly, P2Y1 and P2Y2 receptors, together with A2AR and A2BR expression are upregulated and IL-1β synthesis increased by ATP metabolites and their stable forms. As these purinergic receptors couple to G proteins, in this study the authors suggested that the signaling pathway involved in this process was mediated by phospholipase C (PLC)-IP3 activation or inhibition of AC-cAMP. Therefore, an increase in cytoplasmic Ca^2+^ or a decrease in cAMP levels were sensed by the NLRP3 inflammasome, increasing its activity (243).

## Conclusions and Future Directions

In this review, we have described the relevance of the purinergic system in osteoarthritic cartilage and how MetS components associated with OA influence this system (18, 160). It is well-established that A2AR activity mediated by adenosine is involved in articular cartilage homeostasis maintenance, and is a key modulator during OA (134). Obesity-triggered stimulation of this receptor could contribute to cartilage loss and OA development in obese patients (141).

On the other hand, a more in-depth study of the contribution of nucleotide receptors in OA and MetS is needed to understand better the underlying mechanisms that activate purinergic signaling in the pathogenesis of OA and elucidate confounding data. For example, the P2X7 receptor mediates ATP related inflammation during OA (169, 251), but this receptor exerts an anti-adipogenic effect (148). Therefore, it remains unclear whether the link between obesity and OA might be lie in ATP receptors.

Apart from the activation or inhibition of adenosine/ATP receptors, other components of the purinergic system are involved in OA progression and may be potential modulators of metabolic inflammation. AMPK is an energy sensor involved in inflammation, metabolism and T2D, which is able to adapt cellular metabolism in response to the cellular nutritional and environmental stage (255). AMPK activity is constitutively present in healthy articular chondrocytes and decreases with age and during OA progression (213, 256). AMPK activity has been described to prevent articular cartilage degeneration during mouse aging (257), and is also required to maintain mitochondrial function and prevent OA (213). As we have described earlier in this review, metformin can activate AMPK, resulting in an increase in extracellular adenosine (194). It is known that metformin is beneficial in obese patients with knee OA, but the contribution of AMPK to this effect requires further study.

Another component that needs further analysis is the contribution of ADA to this disease. ADA is an indicator of cellular immunity, and its levels are increased in the following diseases: RA, psoriasis, sarcoidosis, cancer and tuberculosis (258). Synovial ADA fluid measurement, in association with CRP and ESR levels, can distinguish OA from other rheumatic diseases, like RA (186). There is a study in the Indian population that links increased ADA activity with being overweight and obesity (150). This link should be studied in obese patients with OA to understand better the role of ADA as an adenosine level regulator.

As mentioned above, several purinergic receptors are involved in inflammasome activation in different diseases. The study of these mechanisms in the inflammation of OA could be an interesting field of research.

In conclusion, the purinergic system is a key modulator of metainflammation, and its contribution to the pathogenesis of OA opens future therapeutic approaches for the treatment of the disease.

## Author Contributions

AM, GH-B, and RL conceptualized, wrote, and revised the manuscript. PG, AL, JM, and IB-Á were primarily responsible for writing, editing, and revising the manuscript. All authors contributed to the article and approved the submitted version.

## Conflict of Interest

AM has filed a patent for the use of adenosine A2AR agonists to prevent prosthesis loosening (pending) and a separate patent concerning the use of A2AR agonists and agents that increase adenosine levels to promote bone formation/regeneration. RL and GH-B have filed a patent on the use of 6-shogaol in osteoarthritis. The remaining authors declare that the research was conducted in the absence of any commercial or financial relationships that could be construed as a potential conflict of interest.
